# An evaluation of the Swiss staging model for hypothermia using case reports from the literature

**DOI:** 10.1186/s13049-016-0210-y

**Published:** 2016-02-17

**Authors:** T. Deslarzes, V. Rousson, B. Yersin, B. Durrer, M. Pasquier

**Affiliations:** University of Lausanne, Lausanne, Switzerland; Emergency Service, University Hospital Centre, Lausanne, Switzerland; Institute of Social and Preventive Medicine, Lausanne University Hospital, Lausanne, Switzerland; Alpine Rescue Service, Air Glaciers, International Mountaineering and Climbing Federation (UIAA), Lauterbrunnen, Switzerland

**Keywords:** Cardiac resuscitation, Core body temperature, Emergency medicine, Hypothermia

## Abstract

**Background:**

Core body temperature is used to stage and guide the management of hypothermic patients, however obtaining accurate measurements of core temperature is challenging, especially in the pre-hospital context. The Swiss staging model for hypothermia uses clinical indicators to stage hypothermia. The proposed temperature range for clinical stage 1 is <35-32 °C (95-90 °F), for stage 2, <32-28 °C (<90-82 °F) for stage 3, <28-24 °C (<82-75 °F), and for stage 4 below 24 °C (75 °F). However, the evidence relating these temperature ranges to the clinical stages needs to be strengthened.

**Methods:**

Medline was used to retrieve data on as many cases of accidental hypothermia (core body temperature <35 °C (95 °F)) as possible. Cases of therapeutic or neonatal hypothermia and those with confounders or insufficient data were excluded. To evaluate the Swiss staging model for hypothermia, we estimated the percentage of those patients who were correctly classified and compared the theoretical with the observed ranges of temperatures for each clinical stage. The number of rescue collapses was also recorded.

**Results:**

We analysed 183 cases; the median temperature for the sample was 25.2 °C (IQR 22-28). 95 of the 183 patients (51.9 %; 95 % CI = 44.7 %-59.2 %) were correctly classified, while the temperature was overestimated in 36 patients (19.7 %; 95 % CI = 13.9 %-25.4 %). We observed important overlaps among the four stage groups with respect to core temperature, the lowest observed temperature being 28.1 °C for Stage 1, 22 °C for Stage 2, 19.3 °C for Stage 3, and 13.7 °C for stage 4.

**Conclusion:**

Predicting core body temperature using clinical indicators is a difficult task. Despite the inherent limitations of our study, it increases the strength of the evidence linking the clinical hypothermia stage to core temperature. Decreasing the thresholds of temperatures distinguishing the different stages would allow a reduction in the number of cases where body temperature is overestimated, avoiding some potentially negative consequences for the management of hypothermic patients.

## Background

Accidental hypothermia is defined as a trunk or a core temperature of less than 35 °C (95 °F), in the context of exposure to cold [1]. Information about core body temperature is used to make decisions about the management and triage to appropriate hospital of hypothermic patients, including avalanche victims. [1, 2] However reliable measurements of core temperature are not always available, especially in pre-hospital settings [3]. The Swiss staging model of hypothermia, which is based on observations of the vital signs at presentation, allows core temperature to be estimated from clinical indicators only [1] (Table 1). It is specifically adapted for use in pre-hospital or austere environments and the hypothermia guidelines recommend that decisions about management of a hypothermic victim should be based on clinical Swiss staging if accurate core body temperature measurement cannot be obtained [1, 4]. The putative correlation between clinical stage and core body temperature is, however, principally based on cases that have not been published in the peer-reviewed literature, and is therefore subject to limitations [5]. Our aim was to evaluate the accuracy and value of the Swiss clinical staging procedure by comparing measurements of core temperature in published case reports of accidental hypothermia, with predictions based on post hoc Swiss staging using data from case reports.

**Table 1:** Swiss clinical staging of hypothermia

	Brown et al., 2012 [1]	Durrer et al., 2003 [4]	Typical core temperature (°C)
Stage 1	Conscious, shivering	Clear consciousness with shivering	35 to 32
Stage 2	Impaired consciousness, not shivering	Impaired consciousness without shivering	<32 to 28
Stage 3	Unconscious, not shivering, vital signs present	Unconsciousness	<28 to 24
Stage 4	No vital signs	Apparent death	<24

There are minor differences between the original system developed Durrer et al. [4], and the most recent versions [1, 5]. Each clinical stage is associated with an estimate of core body temperature

## Method

We searched MEDLINE to find as many cases of hypothermia (core body temperature <35 °C) as possible. We used the keyword ‘hypothermia’ and limited our search to case reports, without imposing any constraints on language or year of publication (last access: February 1st 2015). Additional relevant articles were hand-searched from references in retrieved publications.

We analysed cases in which core temperature was below 35 °C and for which data on clinical parameters and vital signs at presentation were available (this allowed cases to be classified clinically using the Swiss staging procedure). Cases without sufficient data or with confounding factors, as described below, were excluded. We also analysed cases where there were initially no data on vital signs but the patient had survived after resuscitation, and fatal cases for which medico-legal examination was available and confirmed hypothermia as the cause of death. To ensure that any impairment in consciousness could be firmly attributed to hypothermia alone we excluded cases with any mention or suspicion of one of the following potential confounding factors: acute alcohol or other intoxication, drug overdose, traumatic brain injury and medical conditions that could lead to hypothermia. Cases of therapeutic and neonatal hypothermia were also excluded. A blood alcohol concentration of up to 150 mg/dl was not grounds for exclusion as this is considered the threshold at which signs of altered consciousness may occur [6]. Daily medication use was also tolerated.

The following data were collected: age, sex, vital parameters, first recorded core body temperature, presence of shivering, occurrence of cardiac arrhythmia, causes of accidental hypothermia, rewarming method, hospital survival and neurological outcome.

Consciousness was evaluated using the Glasgow Coma Scale (GCS), the Alert-Verbal-Pain-Unresponsive (AVPU) classification, or any other descriptive clinical information available. Twelve authors were contacted by e-mail to obtain information that was missing from the published report, with 2 additional items of data collected. In view of the very low rate of documentation of shivering in the first cohort of cases collected, we decided to stage hypothermia clinically solely on the basis of state of consciousness and vital signs. Stage 1 was defined as a GCS score =15 or ‘A’ from the AVPU classification; Stage 2 as a GCS score > 8 and < 15 or ‘V’ from AVPU; stage 3 as a GCS < 9 or ‘P’ or ‘U’ from AVPU; Stage 4 as the absence of vital signs (respiratory rate of 0, no measurable blood pressure, no palpable pulse) and GCS = 3 or ‘U’ from AVPU.

### Statistical analysis

The percentage of cases that would have been correctly classified using the Swiss clinical staging procedure was obtained as the number of patients for which the observed temperature lay within the theoretical range of temperature associated to his/her clinical stage. A logistic regression has been carried out to explore whether sex, age, and the cause of the hypothermia influenced the percentage of cases correctly classified. In each stage group, a 95 % prediction interval for the temperature was obtained by adding to and subtracting two standard deviations from the average calculated in that group. As an additional analysis, we performed three receiver operating characteristic (ROC) analyses to determine optimal temperature thresholds for discriminating between the four clinical stage groups. For every possible threshold temperature t we calculated sensitivity as the proportion of patients in the higher stage group with a temperature below t, and specificity as the proportion of patients in the lower stage group with a temperature equal to or above t. Each threshold t can thus be defined in terms of an (x,y) pair (sensitivity, specificity). A ROC curve is a plot of values of sensitivity against one minus specificity. The area under that curve (AUC) is a measure of how well a test variable (in this case temperature) discriminates between two groups. It also represents the probability that a randomly chosen patient from the higher stage group will have a lower temperature than a randomly chosen patient from the lower stage group. The threshold temperature which best discriminates between two group was taken to be the temperature at which the sum of sensitivity plus specificity was maximal (which is related to the Youden index) [7]. 95 % Confidence intervals for these threshold temperatures have been calculated using the bootstrap method.

## Results

**Fig. 1:**
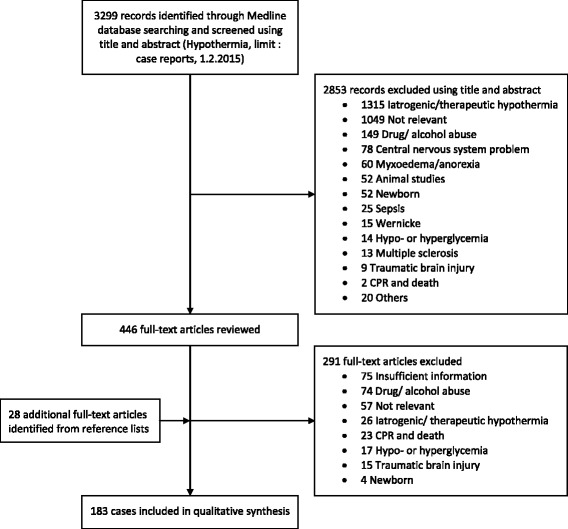
Flowchart of study cases

We identified 183 cases of hypothermia which were suitable for analysis (Fig. 1). Their characteristics are presented in Table 2. Shivering was present in 8 out of 11 patients for whom the information was available. Only 3 of these patients had temperatures corresponding to Stage 1 hypothermia, 2 to Stage 2, and 3 to Stage 3. Only 3 out of the 8 patients with shivering had a GCS score of 15 or ’A’ on the AVPU classification. Among the causes of hypothermia disposable, we found 77 cases of water exposition, 8 cases of avalanche accident and 26 cases of environmental exposure (snow or wind exposure, fall in a glacier crevasse, cool chamber trapping).

**Table 2:** Characteristics of the 183 patients included

Age in years, median (IQR)	40 (17;60)
Sex male, n (%)	111 (61)
Temperature in °C, median (IQR)	25.2 (22;28)
First cardiac rhythm, n (%)	
Asystole	36 (31)
Sinus rhythm	35 (29.9)
Ventricular fibrillation	23 (19.7)
Atrial fibrillation	13 (11.1)
Others/unknown	66 (36)
Rewarming method (information available for 166 patients), n (%)	
ECMO/CPB	62 (37.4)
Haemodialysis	9 (5.4)
Endovascular	5 (3.0)
Others/external rewarming	90 (54.2)
Outcome (CPC available for 125 patients), n (%)	
1	149 (81.4)
2 or 3	10 (5.5)
4	1 (0.5)
5	6 (3.3)
Missing information	17 (9.3)

CPB: cardiopulmonary bypass; CPC [16]: cerebral performance categories (1 = normal or slightly diminished cerebral function, 2 = moderate cerebral disability, 3 = severe cerebral disability, 4 = coma or vegetative state, 5 = brain dead); ECMO: extracorporeal membrane oxygenation; IQR: interquartile range

The classification of patients according to clinical stage and measured core body temperature is presented in Table 3. Ninety-five out of the 183 patients (51.9 %, 95 % CI: 44.7 %-59.2 %) had a core body temperature within the predicted range for their clinical stage, i.e. were correctly classified on the basis of clinical staging, while the temperature was overestimated in 36 patients (19.7 %, 95 % CI: 13.9 %-25.4 %) and underestimated in 52 patients (28.4 %, 95 % CI: 13.9 %-25.4 %). Sex, age, cause and the site of temperature measurement were not significantly associated to the accuracy of clinical staging. The observed mean temperature was 31.3 °C for patients clinically classified as Stage 1, 28.3 °C for those classified as Stage 2, 25.6 °C for Stage 3 and 22.7 °C for Stage 4. We observed important overlaps among the four stage groups with respect to core temperature, the lowest observed temperature being 28.1 °C for Stage 1, 22 °C for Stage 2, 19.3 °C for Stage 3, and 13.7 °C for stage 4. In other words, the observed lowest temperature in the Stage 1 group corresponded to the lowest theoretical temperature for the Stage 2 group, whereas the observed lowest temperature in the Stage 2 and Stage 3 groups were well below the theoretical temperature for the Stage 3 group. Results were quite similar when replacing these lowest observed temperatures with the lowest bound of a 95 % prediction interval based on a normal distribution (see the right column in Table 3).

**Table 3:** Correspondence between clinical stage and the measured temperature for the 183 cases. The increase in the percentage of cases classified correctly at higher stages was globally non-significant in a chi-square test (*p* = 0.48) due to the small number of patients in the Stage 1 and Stage 2 groups. T° = core body temperature in °C

	≥32 T° <35	≥28 T° <32	≥24 T <28	T° < 24	overall, N (%)	mean T ± SD^a^	95 % CI for mean	95 % prediction interval^b^
Stage 1, n (%)	4	6	0	0	10 (5.5)	31.3 ± 2.2	29.7-32.9	26.9-35.7
Stage 2, n (%)	3	11	8	2	24 (13.1)	28.3 ± 3.2	27.0-29.6	22.0-34.6
Stage 3, n (%)	3	12	33	20	68 (37.2)	25.6 ± 3.2	24.9-26.4	19.3-32.0
Stage 4, n (%)	0	9	25	47	81 (44.3)	22.7 ± 4.3	21.7-23.6	14.0-31.4

^a^In nine cases, we retained the lowest temperature of the thermometer as the actual temperature

^b^95 % prediction intervals were calculated assuming normality as mean ± 2SD

**Fig. 2:**
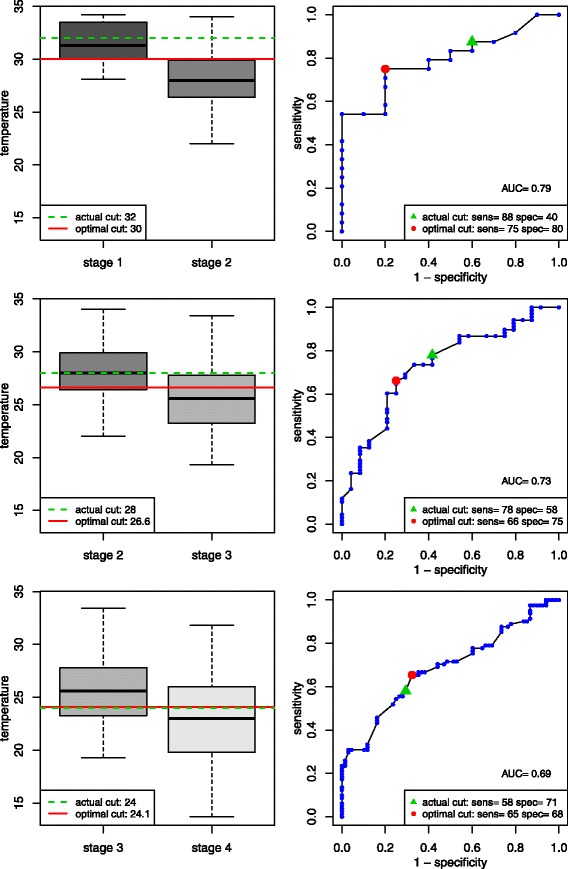
ROC curves and optimal core body temperature thresholds in °C for stage discrimination. The temperature which best distinguishes between stages was taken as the temperature value which maximises the sum of sensitivity plus specificity. Using estimated optimal thresholds increased (sensitivity + specificity) from 128 to 155 for the stage 1/2 threshold, from 136 to 141 for the stage 2/3 threshold and from 129 to 133 for the Stage 3/4 threshold. Actual cut = accepted threshold for current clinical Swiss staging system [4]; Optimal cut = optimal threshold i.e. the temperature at which the sum sensitivity + specificity is maximal; AUC = area under the curve

The results of the ROC analyses are shown in Fig. 2. The optimal temperature threshold for discriminating between stages were as follows, Stage 1 and Stage 2: 30 °C (actual cutoff = 32 °C; 95 % bootstrap CI: 28.1 °C -31.5 °C); Stage 2 and Stage 3: 26.6 °C (actual cutoff = 28 °C; 95 % bootstrap CI: 25.0 °C-29.0 °C); Stage 3 and Stage 4: 24.1 °C (actual cutoff = 24 °C; 95 % bootstrap CI 20.6 °C-25.3 °C).

Rescue collapse occurred in 15 patients, of whom all but 1 were in clinical stage 3 at presentation with either ventricular fibrillation (*n* = 10), or asystole (*n* = 5); the remaining patient was in clinical stage 2 at presentation (core temperature of 22 °C) [8]. The highest temperature recorded in a patient who later suffered rescue collapse was 27.8 °C [9].

## Discussion

### Correct classification rate and potential clinical consequences of misclassification

Using the theoretically derived temperature ranges for clinical stages would result in about 50 % of patients being assigned to the wrong temperature range. The potential consequences of such misclassification are variable; they depend on the scenario considered [1]. Clinically classifying a patient with a core temperature ≥ 32 °C as stage 2 would, at worst, result in more cautious management and monitoring of the patient, in view of the risk of harmful arrhythmias when core temperature drops below 32 °C. Conversely, clinically classifying a patient who actually had a core temperature <32 °C as stage 1 would result in underestimation of the risk of cardiac arrest, with potentially serious consequences. Six of our patients in clinical stage 1 actually had temperatures below the predicted range for stage 1; the lowest temperature recorded from a clinical stage 1 patient was 28.1 °C [10], which is very close to the 28 °C threshold below which the risk of cardiac arrest increases substantially. Clinically classifying a patient with a core temperature ≥28 °C as stage 3 would result in him or her being transported to a hospital capable of providing extracorporeal re-warming rather than to the nearest hospital. Clinically classifying a patient with a core temperature < 28 °C as Stage 2 is probably the most concerning scenario, as it could result in the patient being transported to hospital without the extracorporeal re-warming facilities which are indicated in such cases. This would have been the case for 10 patients in our study, 1 of whom suffered cardiac arrest during transportation [8]. Misclassification of patients with a temperature <24 °C as clinical stage 3 has recently been the subject of investigation; this form of misclassification may result in underestimation of the risk of cardiac arrest, which is extremely high for patients with a core temperature <24 °C [5]. Finally, the cases of cardiac arrest in patients with a temperature ≥24 °C and a favourable outcome are possibly over-reported (publication bias), which could lead to an unrepresentative selection of Stage 4 hypothermic patients with higher core temperature than in reality. Hypothermic patients in cardiac arrest (Stage 4) had an excellent outcome, 61 out of the 71 Stage 4 patient for whom outcome information was available (85 %) having a CPC at 1. Whether the initial cardiac arrest was correctly diagnosed in these patients is also a matter of debate, as minimal vital signs can be easily missed in Stage 3 deeply hypothermic patients, especially if the search is not carried out scrupulously [5, 11].

### Temperature ranges and estimates of optimal temperature thresholds

We showed clearly that there were important overlaps between the four stage groups with respect to core temperature which indicates that accurate diagnosis of hypothermic stage cannot be based solely on temperature. This suggests that it might be preferable to associate the various stages with overlapping temperature ranges, based - for example - on the last column of Table 3. We also note that the average temperature observed in the Stage 1 group was below the theoretical lower boundary for this stage, whilst the average temperature observed in the Stage 2 group was close to theoretical lower boundary; this suggests that a redefinition of the temperature ranges associated with the different clinical stages may be necessary.

### Modifying thresholds of temperatures distinguishing the different stages

As mentioned above, it would be more correct to associate overlapping ranges of temperature, respectively a minimal temperature associated to the different clinical stage groups, rather than to consider thresholds of temperature to distinguish the different stages, set at respectively 32 °C, 28 °C and 24 °C, as done so far. If one would like to keep these thresholds, one may need to study how to modify the definition of the clinical stages to improve the percentage of correct classification, in particular to minimize the proportion of patients for whom the temperature is overestimated. An alternative possibility would be to study how to modify the thresholds of temperatures to improve the situation. Obviously, decreasing the thresholds would minimize the proportion of patients for whom the temperature is overestimated, at the cost of increasing drastically the proportion of patients for whom the temperature is underestimated. Some discussion should take place here to establish what would be the most desirable compromise between underestimation and overestimation of the temperature. It was to explore such possible compromises that we performed a ROC analysis (although technically we had to consider in this analysis the temperature as the predictor and the clinical stage as the outcome since a predictor in a classical ROC analysis has to be a quantitative variable). Our estimates of the optimal thresholds for discriminating between the stage 1 and stage 2 groups and the stage 2 and stage 3 groups were lower than the currently accepted ones (30.0 °C vs. 32 °C and 26.6 °C vs. 28 °C respectively). The currently accepted stage 1/2 threshold does not even fall within the 95 % bootstrap CI for the estimated optimal threshold. In contrast, our estimated optimal stage 2/3 threshold was not significantly lower than the accepted threshold, which falls within the 95 % bootstrap CI for our estimated threshold. Our estimated optimal stage 3/4 threshold was remarkably close to the accepted threshold (24.1 °C vs. 24 °C respectively). Overall core body temperature discriminated best between the Stage 1 and Stage 2 groups (see also the AUC values).

Our estimated thresholds are not too far from the accepted thresholds, although one could decrease the stage1/2 threshold from 32 °C to 30 °C, and the stage 2/3 threshold from 28 °C to 27 °C. The clinical consequence of such changes would be a slight reduction of the percentage of patients for whom the temperature is overestimated (in our data, from 19.7 % to 15.8 %), while keeping the percentage of patients for whom the temperature is underestimated at around 33 %, yielding hence a greater appreciation of the risk of cardiac arrest, which has implications for patient management and the decision about orientating the patient to the most appropriate treatment facility.

### Others

While including the first patients we noted that presence of shivering was very seldom reported. In only 11 cases was this data reported: 8 where shivering was present and 3 where it was absent. Five patients of the eight with shivering were colder than the temperature indicated by clinical staging, and most were young and active. The lowest temperature recorded in presence of recorded shivering was 21 °C (core) in a 44 year-old patient [12]. The ‘reassuring’ nature of the presence of shivering should perhaps be reconsidered, with rescuers considering the possibility of deep hypothermia even in the presence of shivering, and being careful with resuscitation.

### Limitations

Our study suffers from some limitations. An important one is the risk of publication bias. The published cases may not be representative of clinical practice; this suggestion is supported by the high number of favourable outcomes and the relative paucity of moderately hypothermic cases. These low numbers should be taken into account when considering our optimal thresholds estimations. We were nevertheless obliged to rely mainly on case reports, as we required accurate descriptions of clinical status. This method however provides better evidence for the link between Swiss clinical stages and core temperature. Another limitation comes from the reliability of the temperature measurement, which is known to be influenced by the type of probe used or - especially in the pre-hospital setting – by environmental factors [13].

The choice of the GCS and AVPU scores as a method of clinical classification is another potential limitation. The categories ‘A’ and ‘U’ have, however, been shown to correspond reliably to GCS scores of 15 and 3 respectively [14]. There is also some overlap between categories, the median GCS scores associated with ‘P’ and ’U’ responses are 13 and 8, respectively [14, 15]. Using the GCS and AVPU scores produce more reproducible results than using the terms ‘clearly conscious’, ‘impaired consciousness’ and ‘unconscious’ as in the original staging guidelines, and could serve as a common reference for further studies.

We were unable to use the criteria of presence or absence of shivering presence in our analyses, because of the paucity of cases where this information was available. The wide range of temperatures observed in both shivering and non-shivering patients does, however, make us dubious about the discriminative power of shivering, at least as a sole criterion. Further prospective studies or better reporting of the presence or absence of shivering in hypothermia cases reports would enable us to gain a better understanding of the value of this criterion. Finally, as with any retrospective chart review, the quality of the data (including the accuracy of temperature measurement) and the relative sample size could also limit our conclusions.

## Conclusion

Measured core body temperature corresponded to the clinical stage in the Swiss staging model of hypothermia in only about 50 % of the cases. Misclassification that lead to underestimation of actual core body temperature are usually benign and lead only to over-utilization of the resources, whereas overestimation of core body temperature has potentially serious consequences for the patient. The estimated optimal temperature thresholds for the various stages are not too far from the currently accepted thresholds, although it may be appropriate to decrease the thresholds for stage 1/2 and stage 2/3, as we found. This may allow a slight reduction in the number of cases where core body temperature is overestimated, avoiding some potentially negative consequences for the management of hypothermic patients. However, it would probably be more sensible to associate overlapping ranges of temperatures to the four groups defined by the Swiss staging procedure, whereas the most cautious and safe approach would be to use the lower published temperature for each clinical stage. Identifying clinical indicators allowing a better prediction of the core body temperature would also be useful given the limitations on field temperature measurement. A better reporting of clinical signs by retrieval teams, or at best dedicated prospective studies would be therefore helpful to better predict core body temperature from the clinical state of hypothermic patients.
